# Enhanced mitochondrial membrane potential and ATP synthesis by photobiomodulation increases viability of the auditory cell line after gentamicin-induced intrinsic apoptosis

**DOI:** 10.1038/s41598-019-55711-9

**Published:** 2019-12-17

**Authors:** So-Young Chang, Min Young Lee, Phil-Sang Chung, Sehwan Kim, Bernard Choi, Myung-Whan Suh, Chung-Ku Rhee, Jae Yun Jung

**Affiliations:** 1 Beckman Laser Institute Korea, Dankook University, 119, Dandae-ro, Dongnam-gu, Cheonan, Chungnam 31116 Republic of Korea; 2 Department of Otolaryngology-Head and Neck Surgery, College of Medicine, Dankook University, 119, Dandae-ro, Dongnam-gu, Cheonan, Chungnam 31116 Republic of Korea; 3 Department of Biomedical Engineering, College of Medicine, Dankook University, 119, Dandae-ro, Dongnam-gu, Cheonan, Chungnam 31116 Republic of Korea; 4 Beckman Laser Institute Korea, Dankook University, Dankook, Republic of Korea; 5 Beckman Laser Institute and Medical Clinic, University of California, Irvine, CA 92612 USA; 6 Department of Otorhinolaryngology-Head and Neck Surgery, Seoul National University Hospital, 101 Daehak-ro, Jongno-gu, Seoul, 03080 Republic of Korea

**Keywords:** Inner ear, Hair cell

## Abstract

Photobiomodulation (PBM) has been suggested to have a therapeutic effect on irreversible hearing loss induced by aminoglycosides, including gentamicin (GM). However, its intracellular mechanism(s) in GM-induced ototoxicity remain poorly understood. In the present study, we investigated the effect of PBM in GM-induced ototoxicity in auditory cells. We tried to characterize the downstream process by PBM, and the process that triggered the increased cell viability of auditory cells. As a result, the effects of PBM against GM-induced ototoxicity by increasing ATP levels and mitochondrial membrane potential was confirmed. These results suggest a theory to explain the therapeutic effects and support the use of PBM for aminoglycoside-induced hearing loss.

## Introduction

Aminoglycosides, which were first developed in the 1940s, are anti-Gram negative bacterial agents that are used worldwide, including in many developing countries, due to their low cost and high efficacy^1,2^. However, they have significant side effects, such as cochlear and vestibular toxicity and nephrotoxicity^3,4^. In particular, permanent hearing loss and/or balance malfunction can result from aminoglycoside treatment, reducing the patient’s quality of life due to lack of communication and social maladjustment. The primary mechanism of inner ear damage from aminoglycosides, such as gentamicin (GM), is considered to be related to apoptosis, specifically through the intrinsic pathway of apoptosis, in which many signals are triggered in the mitochondrial pathway^5^. However, the detailed mechanism is still unclear, and many on-going studies are still being conducted. Thus, various therapies, such as the administration of steroids, antioxidants, and free radical scavengers, have been investigated to ameliorate the ototoxic damage caused by aminoglycosides^6–10^. However, the clinical applicability of these approaches remains largely unclear.

In modern medicine, light therapy is applied in various fields. Photobiomodulation (PBM) uses an output power of 1–500 mW, which does not induce a temperature increase in any tissues or cells, and mostly includes the red and near-infrared range of light, with wavelengths of 600–1,000 nm^11–14^. Previous studies from our laboratory have reported the effects of PBM in GM-induced ototoxicity^15,16^ both *in vitro* and *in vivo*. Although PBM has been reported to have beneficial effects in medical applications, the mechanisms of PBM have not been fully understood. The prevailing theory regarding the mechanism of PBM involves bio-stimulation of mitochondria induced by laser irradiation^17–19^.

In the present study, we first determined the increased cell viability after PBM in GM-induced ototoxicity in auditory cells. We characterized the reverse of the downstream process of apoptosis by PBM, and then characterized the process that triggered the increase of auditory cell viability by analyzing serial levels of ATP and mitochondrial membrane potential (MMP) immediately after PBM in GM-induced ototoxicity.

## Results

### Characteristics of HEI-OC1 cells and confirmation of GM-induced toxicity

**Figure 1:**
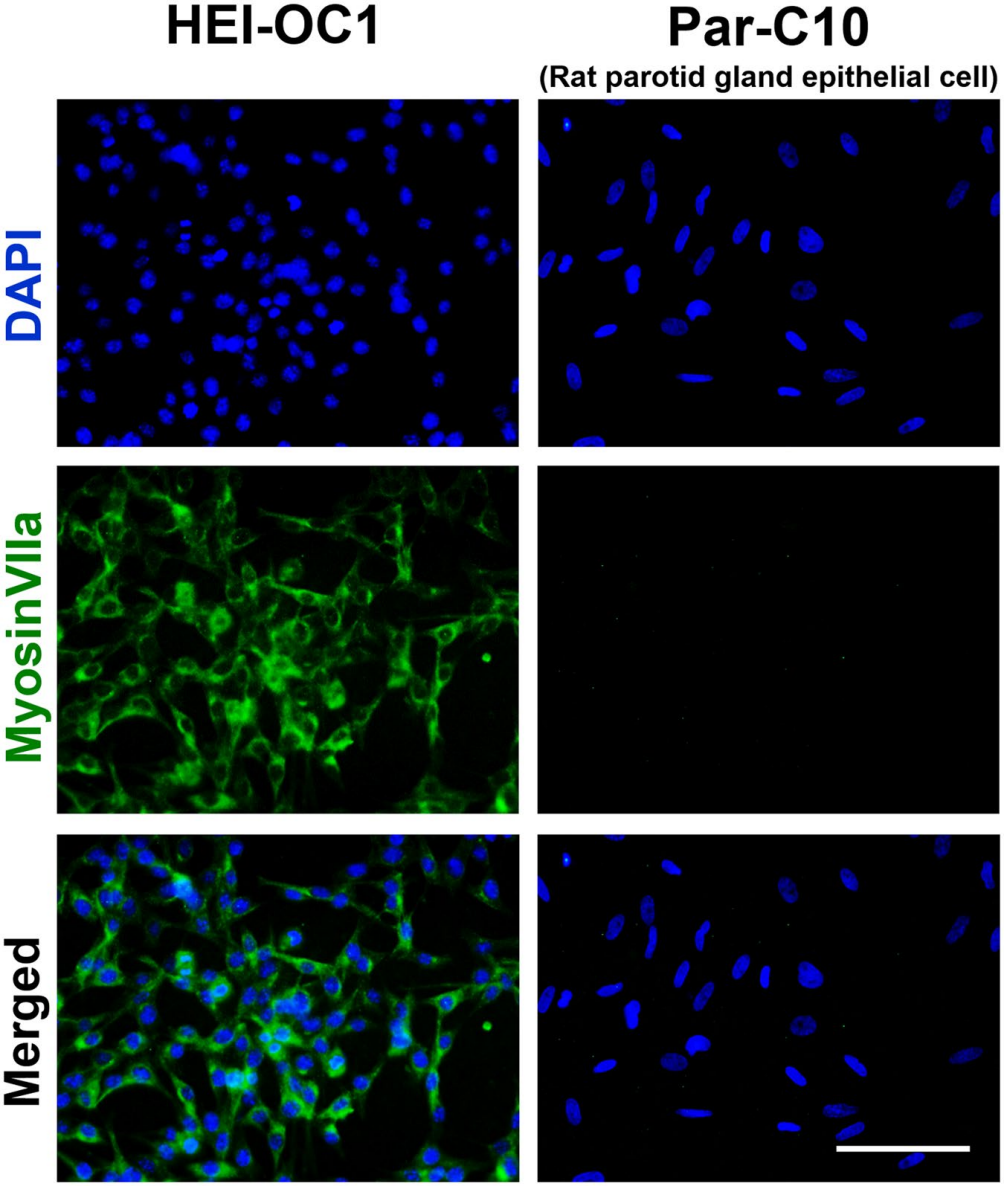
Epifluorescence analysis of HEI-OC1 cells. Cell nuclei were stained with 4′,6-diamidino-2-phenylindole (DAPI), with both cell lines well stained (blue). Myosin VIIa was used as an inner ear hair cell marker. HEI-OC1 cells were positive for myosin VIIa (green); the Par-C10 cells (rat parotid gland epithelial cells) were used as a negative control and were not stained (scale bar: 100 µm).

To determine whether House Ear Institute-Organ of Corti 1 (HEI-OC1) cells could serve as a model of cochlear hair cells, we conducted epifluorescence analyses using myosinVIIa staining. Par-C10 cells, a rat parotid gland cell line, were used as a negative control. HEI-OC1 cells stained for myosinVIIa, whereas Par-C10 cells did not (Fig. 1). Based on these results, we assumed that HEI-OC1 cells could be used as an *in vitro* model of cochlear hair cells.

**Figure 2:**
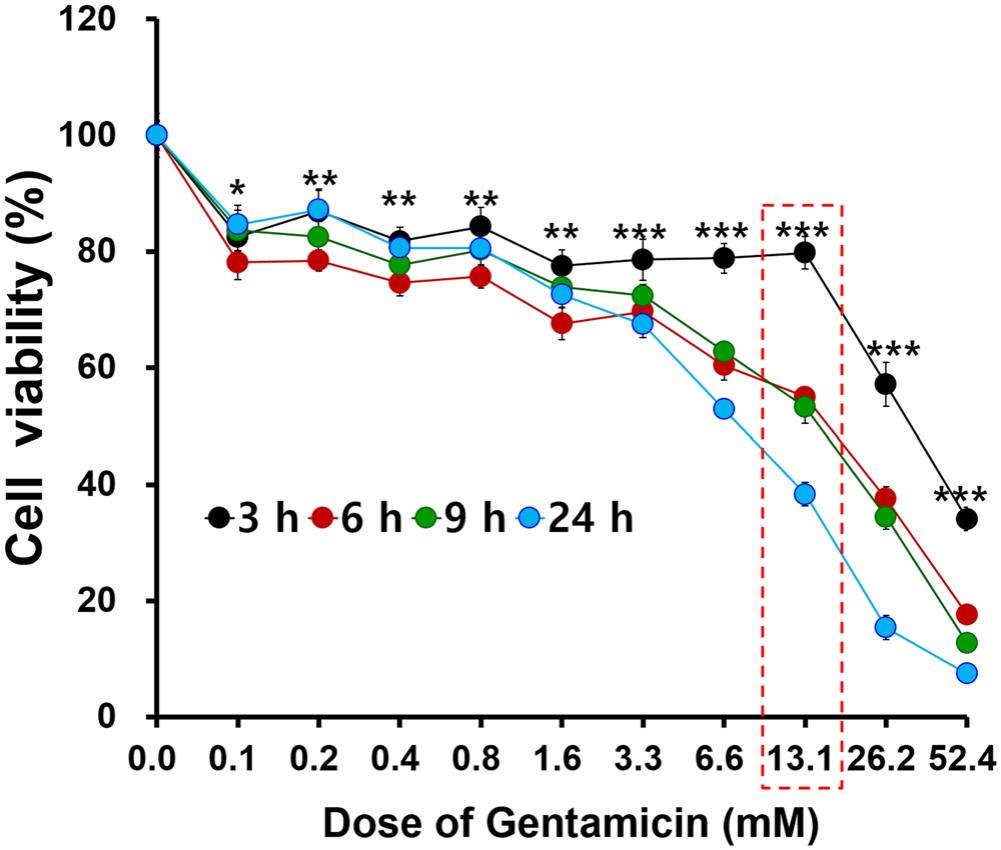
Cell viability after treatment with different gentamicin (GM) concentrations for different time periods; determination of GM concentrations for experimental evaluations. The cell viability of GM-treated HEI-OC1 cells decreased with increasing GM concentrations. The four differently colored lines indicate the cell viabilities at four different time points (3 h, 6 h, 9 h, and 24 h after GM exposure). At all GM concentrations, the cell viabilities differed significantly among the four time points (p-values between 0.05 and <0.001). The cell viability at 24 h decreased and was <50% at a concentration of 13.1 mM. Furthermore, the largest cell survival difference between the 3 h and 24 h time points was observed at this concentration. A GM concentration of 13.1 mM (red dotted rectangle) was therefore used for further studies.

To assess the possibility that HEI-OC1 cells might be damaged after GM inoculation, cell viability after GM treatment was assessed. Cell viability decreased in a dose-dependent manner. Significantly different cell viabilities among the time points were found using GM concentrations >0.1 mM (detailed statistics in Table 1). These results suggested that HEI-OC1 cells were damaged by GM and that its toxicity was dose and time dependent. Massive cell death occurred with concentrations >26.2 mM, and the largest cell viability difference among time points was observed at a GM concentration of 13.1 mM (Fig. 2). We determined that the optimal concentration for the present study would be 13.1 mM based on these results.

**Table 1:** Statistical analysis of cell viability after GM treatment.

Concentration (mM)	1way ANOVA (P value)	3 vs 6 h	3 vs 9 h	3 vs 24 h	6 vs 9 h	6 vs 24 h	9 vs 24 h
0.0	ns	ns	ns	ns	ns	ns	ns
0.1	0.0252 (*)	ns	ns	ns	ns	*	ns
0.2	0.0016 (**)	**	ns	ns	ns	**	ns
0.4	0.0029 (**)	**	ns	ns	ns	*	ns
0.8	0.0041 (**)	**	ns	ns	ns	ns	ns
1.6	0.0018 (**)	**	ns	ns	ns	ns	ns
3.3	0.0008 (***)	*	ns	***	ns	ns	ns
6.6	0.0002 (***)	*	ns	***	ns	ns	*
13.1	0.0003 (***)	ns	ns	***	ns	ns	ns
26.2	0.0001 (***)	ns	*	***	ns	*	ns
52.4	<0.0001 (***)	ns	*	***	ns	*	ns

### Increased HEI-OC1 cell viability by PBM and determination of the optimal PBM time point

**Figure 3:**
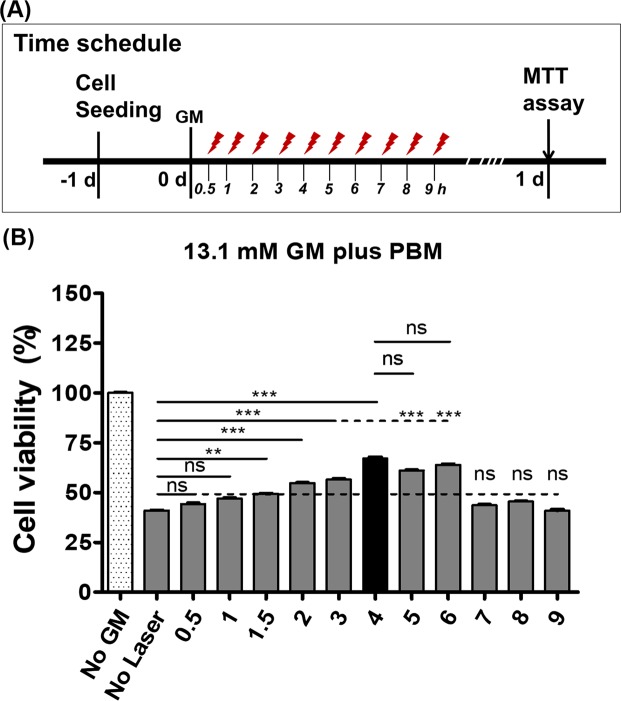
Determination of the optimal photobiomodulation (PBM) time after gentamicin (GM) exposure for the rescue of cell viability against GM toxicity. Different PBM times are shown in (**A**). PBM (15 min duration, power of 15 mW) was used for irradiation for 0.5–9 h after GM exposure, and the cell viability was determined at 24 h after GM exposure using the MTT assay. As explained in Fig. 2, a GM concentration of 13.1 mM was used for this study. Cell viabilities were different among variable PBM time points. Significant increases in cell viability were observed with post hoc analysis using PBM for 1.5–6 h after GM exposure (**B**). The highest recovery of cell viability was observed with laser irradiation for 4 h after GM exposure. (*p < 0.05; **p < 0.01; ***p < 0.001).

PBM (15 mW, 15 min) was performed at various time points (Fig. 3A), from immediately (0 h) to 9 h after GM treatment. Up to the 6-h time point, cell viability increased significantly compared with the control group, and PBM was most effective at 4 h after GM treatment (Fig. 3B; one-way analysis of variance using the Kruskal–Wallis test, KW = 278.3, p ≤ 0.0001; n = 20–24; post hoc Dunn’s test, p < 0.001~p < 0.0001). These results suggested that PBM partially repopulated HEI-OC1 cells from GM toxicity and that the PBM initiation time point after GM exposure was a crucial factor. We used this PBM time point of 4 h for further experiments to observe the downstream pathway of apoptotic cell death, which has been suggested to be the mechanism of GM cytotoxicity^20^.

### Confirmation of caspase activation by GM and deactivation by PBM

**Figure 4:**
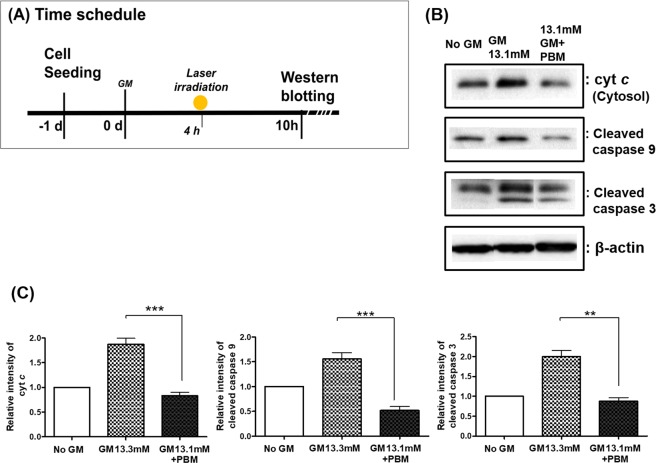
Changes in downstream proteins of the intrinsic apoptotic pathway after gentamicin (GM) exposure with and without photobiomodulation (PBM). (**A**) Proteins related to the intrinsic apoptotic pathway were evaluated by Western blot analysis at 6 h after PBM (10 h after GM exposure). (**B**) The intensities of cytochrome c, cleaved caspase 9, and cleaved caspase 3 expression were increased by GM exposure and were not increased by GM exposure + PBM. Shown blots were cropped to see easy, and the full-length blots were presented in Supplementary Fig. 1. (**C**) Quantification of these data showed differences among groups, and all proteins were statistically higher in the GM exposure group compared to the GM + PBM group (post hoc analysis) (**p < 0.01; ***p < 0.001).

We investigated changes in downstream proteins, such as cytochrome c (Cyt c), that are released by permeabilization of mitochondria, and caspases, which initiate the caspase cascade^21^. Western blotting showed that, at 6 h after laser irradiation (Fig. 4A, all analyzed protein (Cyt c, cleaved caspase 9, and cleaved caspase 3) levels increased only in GM-treated cells. However, in the GM + PBM-treated cells, the proteins were significantly reduced. Displayed blots were cropped, and full-length blots were presented in Supplementary Fig. 1. (Fig. 4B). In addition, there relative intensity differences in the Western blot analyses were observed among different groups (no GM, only GM, and GM + PBM) in all three proteins (one-way analysis of variance using the Kruskal–Wallis test: Cyt c: KW = 14.37, p = 0.0008; cleaved caspase 9: KW = 14.63, p = 0.0007; cleaved caspase 3: KW = 10.83, p = 0.0045). Using post hoc analysis, GM + PBM treatment reduced all protein intensities compared to GM treatment only (all proteins: Dunn’s test, p < 0.01~p < 0.001). These results suggested that Cyt c- and caspase-dependent apoptosis were inhibited by the use of PBM after GM.

### Evaluation of serial ATP levels after PBM

**Figure 5:**
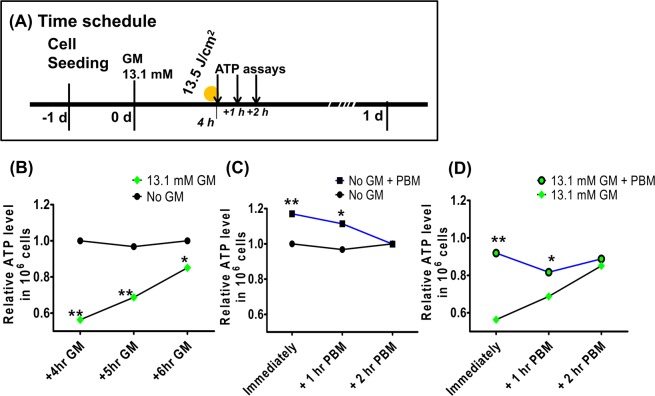
Serial ATP evaluation following photobiomodulation (PBM). ATP levels were measured at 0 h, 1 h, and 2 h after PBM, i.e., 4 h, 5 h, and 6 h after gentamicin (GM) exposure. (**A**) The ATP assay time schedule. GM exposure led to reduction in ATP levels. GM-exposed cells showed significantly different ATP levels compared to cells without GM exposure at all analyzed times (post hoc analysis) (**B**). In cells that were not exposed to GM, after PBM, ATPs levels were significantly different. At the early stage of PBM (0 and 1 h after PBM), ATP levels were significantly elevated in the PBM groups compared no PBM (post hoc analysis) (**C**). After PBM of GM-treated cells, the ATP levels were significantly different in the GM-PBM group compared to the GM only group. (**D**) Also, at early stages of PBM (0 and 1 h after PBM), ATP levels were significantly elevated in the GM + PBM group compared with the GM only group (post hoc analysis). (*p < 0.05; **p < 0.01; ***p < 0.001).

ATP is required for cell survival and metabolism, and GM-induced ototoxicity is known to be associated with a decrease in intracellular ATP. We therefore evaluated ATP changes after GM exposure, and also evaluated alterations in these ATP changes by PBM (Fig. 5A). After GM treatment, the ATP level showed significant decrease compared to control without GM at all time points (Mann–Whitney U-test, two-tailed, n = 8; 4 h after GM: p = 0.0021, U = 0.0; 5 h after GM: p = 0.0024, U = 0.0; 6 h after GM: p = 0.0192, U = 2.0) and showed a time-dependent increasing tendency (Fig. 5B). The increase in ATP was observed immediately and at 1 h after PBM treatment of HEI-OC1 cells with no drug treatment (Mann–Whitney U-test, two-tailed, n = 8; immediately after PBM: p = 0.0042, U = 0.0; 1 h after PBM: p = 0.0402, U = 5.5). These results corresponded to previous reports showing that GM ototoxicity was related to ATP reduction and suggested that PBM increased the ATP level in control auditory cells (Fig. 5C). In a combination of these two experiments, ATP levels were compared only between GM exposed cells and sequential GM + PBM cells, which were serially exposed. Significantly different ATP levels were observed immediately and at 1 h after PBM treatment, indicating higher ATP levels in the GM + PBM group relative to the PBM only group (Mann–Whitney U-test, two-tailed, n = 8; immediately after PBM: p = 0.0080, U = 0.0; and 1 h after PBM: p = 0.0122, U = 7.0) (Fig. 5D). Together, these results showed that ATP levels increased by PBM treatment were still effective during GM toxicity and that PBM reversed the GM-related decrease in ATP.

### Reversal of PBM cell rescue by an ATP blocker

**Figure 6:**
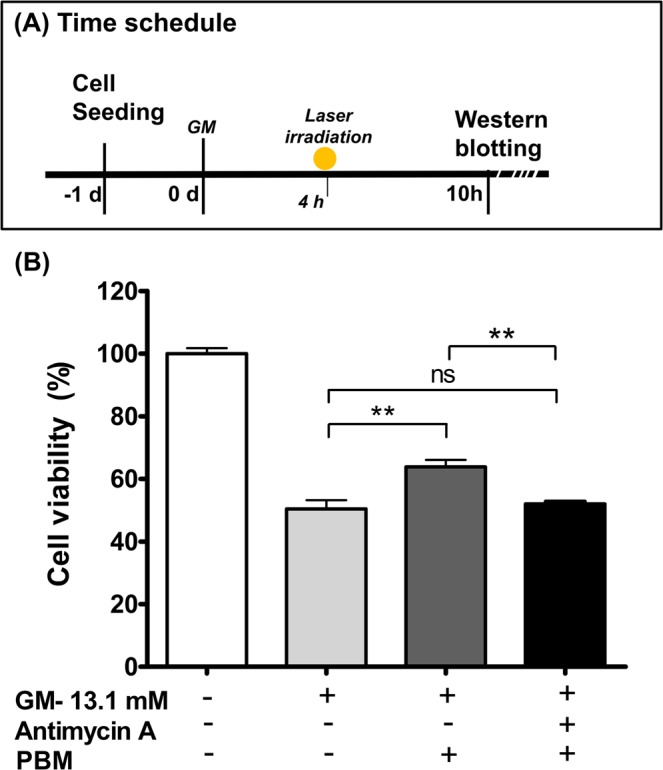
Alteration of cell viability by antimycin A (ATP blocker) during the rescue process of photobiomodulation (PBM) of gentamicin (GM)-treated HEI-OC1 cells. An ATP blocker, antimycin A, was used to reverse the effect of PBM against GM toxicity of HEI-OC1 cells. A. Antimycin A was applied immediately after GM exposure until the initiation of PBM. B. Cell viability of GM only, GM + PBM, and GM + PBM + Amtimycin A groups were compared [Control (no GM and no PBM) is demonstrated in figure but excluded for statistical analysis]. Significantly lower cell viability was observed in antimycin A- and PBM-exposed cells compared to the cells not exposed to antimycin A. (**p < 0.01).

To confirm that an increase in ATP levels^2^ played an important role during the rescue process of PBM against GM toxicity, an ATP blocker was applied prior to laser irradiation (Fig. 6A). Specifically, a 4-h treatment of 0.1 μM antimycin A was applied; at this concentration and time, the potential for cell damage by ATP depletion was small (Supplementary Fig. 2). After PBM application, compared to the group without antimycin A, the antimycin A treatment group showed significantly reduced cell viability. There was no significant difference between GM only and GM + antimycin A + PBM (one-way ANOVA p = 0.0010; F = 13.74; df = 13; n = 4–5; Tukey’s Multiple Comparison Test, GM vs GM + PBM p < 0.01; GM + PBM vs GM + PBM + AA p < 0.01) (Fig. 6B). These results showed that without ATP, the rescue effect of PBM was significantly diminished, supporting the importance of ATP as a key factor for the effect of PBM.

### Indirect assessment of mitochondrial membrane potential after PBM

**Figure 7:**
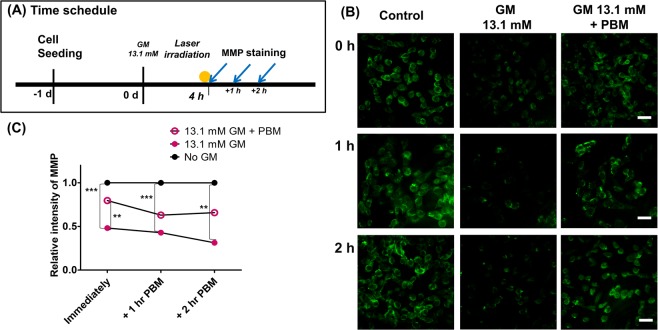
Serial mitochondrial membrane potential (MMP) changes after laser irradiation in a gentamicin (GM) ototoxicity model. (**A**) MMP intensity was measured using 10 mM rhodamine 123 staining at 0 h, 1 h, and 2 h after photobiomodulation (PBM). A. As shown in the images, MMP expression was dramatically reduced in the GM groups (middle column) compared to the control (no GM; left column) at all time points (all three rows). (**B**) These results were reversed by PBM exposure, and MMP expressions increased in the GM + PBM group (right column) compared to the GM only group (middle column); it then returned to control levels (left column). (**C**) Quantification of these data showed differences among groups, with higher MMP in the GM exposure group compared to controls at all time points. Immediately after PBM, the GM + PBM group showed significantly higher MMP expression compared to the GM only group (post hoc analysis). (**p < 0.01; ***p < 0.001; scale bar: 100 µm).

We evaluated changes in Rhodamine 123 by its epifluorescence intensity to monitor the membrane potential of mitochondria^22^ at the a treatment when compared to controls, whereas it was significantly increased after PBM (Fig. 7B). Using these epifluorescence images, MMP was quantified and compared among groups (control, only GM, and GM + PBM) at three time points (immediately, 1 h, and 2 h after PBM). Differences among groups were observed at all time points (one-way analysis of variance with the Kruskal–Wallis test: immediately: KW = 24.76, p < 0.0001; 1 h: KW = 16.54, p = 0.0003; and 2 h: KW = 12.10, p = 0.0024). Post hoc analysis revealed that GM treatment reduced MMP at all time points (Dunn’s test, p < 0.01~p < 0.001). Post hoc analysis showed a significant increase only immediately after PBM (Dunn’s test, p < 0.01) (Fig. 7C). This timely PBM response of MMP led us to predict that light energy would affect MMP following further increases in ATP levels.

## Discussion

Although PBM, among several phototherapeutic devices, has been approved by the US Food and Drug Administration, no theoretical background has been fully established. This study suggested a theoretical basis for the clinical application of PBM. After the report that revealed relation of nucleic factor kappaB and auditory cell damage by acoustic overexposure by Tamura *et al*.^23^, this is the following report to analyze how PBM affects a pathway related to inner ear ototoxicity. This information could help to facilitate the efficient use of appropriate laser techniques and therapies (e.g., molecular) to enhance its efficacy. Finally, with a theoretical background, PBM as a protective measure could potentially be used with drugs that are inevitably ototoxic in clinical settings.

There are two major flows in hearing research. One is the regeneration of missing hair cells and the other is the rescue of damaged hair cells. According to previous studies, PBM seems to affect both processes of hearing recovery. Rhee, *et al*. suggested that PBM effect on gentamicin induced ototoxicity may be an impaction of cells proliferation and/or transdifferentiating^15^. One the other hand, there is another study by Rhee, *et al*. in the same year assessing protective effect of PBM after ototoxicity^24^. Therefore it might be necessary to clarify whether the pathway that revealed in our study is related to proliferation/transdifferentiation or protection. HEI-OC1 cell line, as explained above, is a common progenitor cell line for hair cell markers and supporting cells of the organ of Corti. It expresses hair cell markers such as Prestin, Myosin VIIa, Atoh1, BDNF, Calbindin and Calmodulin and supporting cell markers like connexin 26 and fibroblast growth factor receptor (FGF-R)^25^. Considering this potency, there is chance that the HEI-OC1 cells could have been repopulated or regenerated by laser treatment after damage rather than protected from GM. Increased cell viability and related markers after PBM without any ototoxic insults indirectly supports the regeneration theory (Supplementary Fig. 3). On the other hand, there are findings in this study which reflected protection or rescue, as proteins that were responsible for the intrinsic apoptotic pathway were downregulated after PBM. Thus, it is not feasible to conclude at this point and we believe that both inhibition of apoptosis and proliferation/regeneration occurred after PBM and increases of ATP/MMP take an important role in this process.

Although we determined the ototoxic concentration by the actual damage model using HEI-OC1 cells, concentration of ototoxic drug applied is this study is beyond the clinical applicability. The reason for this might be related to the difference of cell structures. In organ culture or *in vivo*, the pathway through which GM enters the hair cell is thought be related to the Mechano-electrical transduction channel (MET channel) located the tips of stereocilia on hair cells, resulting in an abnormal rise in reactive oxygen species (ROS) and initiating cell death^26^. Unlike organ cultures or *in vivo* studies with MET channels, HEI-OC1 cells do not have a MET channel through which aminoglycosides can migrate. Therefore, high drug concentrations may be necessary for drugs to be toxic. Indeed, some researchers have been studied using high concentrations of drug for GM ototoxicity^27,28^.

Many published reports regarding laser therapy include references to optimal parameters, i.e., wavelength, intensity, and duration, for deriving a “good” effect; however, because PBM has frequently shown a biphasic response with different parameters, such recommendations could result in undesirable outcomes^19,29^. Laser parameters should therefore be determined carefully before actual use. In the present study, the wavelength and laser power values were determined from our previous reports and from preliminary studies. Regarding wavelength, we used 808 nm, which is widely used for protective studies of cochlea damage because of its deep penetration^15^. We used a laser power of 15 mW, which was calculated when considering the laser penetration of the inner ear (5%)^16^ and the safety range of laser power in transcanal laser use (<250 mW)^30^. The duration of laser exposure was determined after evaluating the most effective exposure duration, which was 15 min (Supplementary Fig. 4).

During the process of apoptotic cell death by GM treatment, PBM exposure upregulated ATP, which is important for cell homeostasis and cell proliferation/differentiation. ATP, which is a trigger molecule used to identify a mechanism for PBM, is one of the major mitochondrial products. Investigating changes in mitochondrial function after PBM would therefore be necessary because MMP is a key parameter for mitochondrial function^31^. In the present study, mitochondrial function, as evaluated by mitochondrial membrane potential, was reduced after GM exposure, but returned to a normal range after PBM, suggesting that mitochondrial function loss was upregulated by PBM. Considering prior studies showing alterations of MMP after PBM^32–34^, this theory is plausible. However, detailed connections and a molecular mechanism connecting PBM and MMP have yet to be identified. Cyt c is a likely candidate protein, as one of the prevailing mechanisms of PBM is thought to be related to a Cyt c-induced increase in ATP production via effects in the mitochondrial respiratory chain^18,35–38^. However, further molecular work is necessary to confirm this theory.

In the present study, PBM increases ATP immediately after exposure. But it reaches almost down to control level after 2 hours of exposure and there is no difference between GM and GM + PBM group at this time point. Similar results have been reported by a study showing ATP increase in the cochlear neuron after near-infrared light irradiation. According to the speculation made in this paper, the possible reason for this could be due to fast ATP consumption of cells in injury conditions^39^. Considering the fact that ATP difference between non-PBM and PBM group is dramatically reduced at 1 days after in GM treated group (not prominent in no GM treated group) in the present study as shown in Fig. 5 and supplementary Fig. 5, it is probable that reduction of ATP difference between PBM group and no-PBM group over time could be related to the insult induced ATP consumption.

In this study, we demonstrated the increased cell viability of auditory cells by PBM after GM-induced ototoxicity, which can cause permanent hair cell damage and sensorineural hearing loss, by increasing ATP levels and MMP. Moreover, we showed that PBM might be related to down regulation of apoptosis via an intrinsic caspase-dependent pathway. These results suggest a theory to support the use of PBM as a therapeutic tool for aminoglycoside-induced hearing loss. However, further studies to establish detailed connections between PBM and MMP are necessary to confirm our findings.

## Materials and Methods

### Cell culture

House Ear Institute-Organ of Corti 1 (HEI-OC1), a mouse auditory cell line^40^, was cultured continuously in high glucose Dulbecco’s Modified Eagle’s medium (DMEM; Corning, Tewksbury, MA, US), supplemented with 10% (v/v) heat-inactivated fetal bovine serum (FBS; Equitech-Bio, Kerville, TX, USA), and was maintained at 33 °C in a humidified incubator under 5% CO_2_ (Thermo Fisher Scientific, Waltham, MA, USA) in air, for non-permissive conditions. The cells were divided into control (no GM and no PBM), PBM only, GM only, and GM + PBM groups. Par-C10 cells as a negative control were prepared as described in Dr. R. Biswas’s report^41^. Briefly, Par-C10 cells were cultured in Dulbecco’s Modified Eagle Medium: Nutrient Mixture F-12 (DMEM/F-12; Corning, Tewksbury, MA, US) supplemented with 10% (v/v) FBS and 1% Penicillin-Streptomycin (Hyclone, Kremplstrasse, Pasching, Austria) at 37 °C in 5% CO_2_ incubator. The cultured cells were fixed for 10 min in 1% paraformaldehyde (Sigma-Aldrich, St. Louis, MO, USA) in cold (−20 °C) methanol (Daejung, Siheung, Republic of Korea).

### The cell viability assay after GM exposure

HEI-OC1 cells were incubated in 96-well plates (SPL, Pocheon, Republic of Korea) at a density of 1 × 10^4^ cells/100 μL for 24 h with fresh medium changed to medium with GM (Sigma-Aldrich, St. Louis, MO, USA, Cat# G1397). For GM exposure groups, different doses of GM solution were used starting at 50 mg/mL, followed by two-fold serial dilutions using culture medium. The control cells were treated with culture media as a vehicle that the same used to prepare gentamicin diluted solution. The cells were then incubated at 33 °C in 5% CO_2_ for up to 24 h. The dose-dependent effects of GM were assessed using the 3-(4, 5-dimethylthiazol-2-yl)-2, 5-diphenyltetrazolium bromide (MTT) assay (Sigma-Aldrich). For the assay, 50 μL MTT solution (2 mg/mL) was added to each well. The plates were then incubated for 4 h at 33 °C under 5% CO_2_. The medium in each well was removed, and the purple formazan crystals were dissolved in 150 μL dimethyl sulfoxide (Junsei Chemical, Tokyo, Japan). After 20 s of shaking the plates in a microplate mixer (Amersham, Buckinghamshire, UK), the optical density (OD) was measured with a microplate reader (*EASY*UVM340; Biochrom, Cambridge, UK) at 540 nm. To assessed the cell viability, number of replicates for each experiment was performed three times with the same samples. Cell viability was calculated using the following formula: cell viability (%) = (mean OD in treated wells/mean OD in control wells) × 100.

### PBM protocol

A near-infrared diode laser (WonTech, Daejeon, Republic of Korea) with a wavelength (continuous wave) of 808 nm was used to irradiate the GM + laser and laser only samples. The target monolayer of cells was irradiated directly at an intensity of 15 mW for 15 min (total energy density: 13.5 J/cm^2^) starting at 4 h after GM treatment (determined by serial testing from 0.5 h to 9 h) as stated in the Fig. 3 and supplementary Fig. 4. The power of the laser was measured at the bottom of an empty plate before laser irradiation of cells with a SOLO2 laser power meter (Gentec-EO, G2E 5N7, Quebec, Canada) and a XLP12-1S-H2-DO detector head (Gentec-EO, G2E 5N7).

### Western blotting

The proteins were extracted using RIPA lysis buffer [50 mM Tris-HCl, 150 mM NaCl, 0.5% Na-deoxycholic acid, 0.1% sodium dodecyl sulfate, and 1% NP-40 containing protease and phosphatase inhibitors (1:100)] (all reagents were purchased from Sigma-Aldrich) and incubated overnight at −20 °C. The cell lysates were collected by centrifugation at 15,000 rpm for 20 min at 4 °C. Cytosolic extracts were obtained from the sample cells using cytoplasmic extract buffer (10 mM HEPES, 10 mM KCl, 0.1 mM EDTA, 0.1 mM EGTA, 1 mM dithiothreitol, and 0.5 mM phenylmethanesulfonyl fluoride) containing protease and phosphatase inhibitors (1:100) (all reagents were purchased from Sigma-Aldrich) and kept on ice for 15 min. Then, 2.5% NP-40 was added, and the mixture was vigorously vortexed. The cell lysates were collected by centrifugation at 12,000 rpm for 5 min at 4 °C. The supernatants were collected and stored at −20 °C until use in the protein assay. The protein concentrations were calculated using a Pierce BCA protein assay kit (Thermo Fisher Scientific). A total of 30 μg of loaded protein was subjected to sodium dodecyl sulfate-polyacrylamide gel electrophoresis using a 12% separation gel. Separated proteins were electrotransferred onto polyvinylidene fluoride membranes (Bio-Rad, Hercules, CA, USA). The blots were blocked with blocking buffer containing 5% bovine serum albumin (BSA; Santa Cruz Biotechnology, Dallas, TX, USA) and 1% Tween-20 (Sigma-Aldrich) in Tris buffer (TBS) for 1 h and then incubated at 4 °C overnight with primary antibodies, following the recommended dilution range of 1:1,000–1:2,000. The following primary antibodies were from Cell Signaling Technology (Danvers, MA, US): Cyt c, caspase 9 (#9509), and caspase 3 (#9662). Membranes were washed with 0.1% TBST for 15 min, then incubated for 1 h at room temperature with horseradish peroxidase-conjugated goat anti-rabbit IgG (sc 2004; Santa Cruz Biotechnology), diluted in TBST (1:2,000), and then washed. Membranes were developed with an Enhanced Chemi Luminescence kit (GE Healthcare, Buckinghamshire, UK), and images were captured using a Kodak *in vivo* image analyzer (Eastman Kodak, Rochester, NY, USA) and Fuji Medical X-ray film (Fujifilm, Tokyo, Japan). (number of replicates for each experiment was three) The intensities of protein bands were assessed by ImageJ software, and histograms of relative protein expressions were prepared^42^.

### Measurement of ATP levels

To measure ATP levels, the Colorimetric ATP assay kit (Abcam, Cambridge, UK) was used according to the manufacturer’s instructions. Briefly, the cells were trypsinized, centrifuged at 1,200 rpm for 5 min, and then collected at 0 h, 1 h, and 2 h after laser irradiation and resuspended in lysis buffer provided with the ATP assay kit. (number of replicates for each experiment was three) The ATP level was measured by a microplate reader using an absorbance at 540 nm. To assess the reversal of PBM cell rescue by an ATP blocker, antimycin A was dissolved it in 50 mg/mL with 95% ethanol and diluted with culture medium.

### Epifluorescence analysis involving the phenotype of HEI-OC1 cells and an evaluation of MMP

For the determination of HEI-OC1 cell characteristics, the primary antibody (rabbit myosin VIIa, 1:200; Proteus Biosciences, Ramona, CA, USA) in blocking solution composed of 1% BSA and 10% goat serum in 1 × phosphate-buffered saline (PBS, pH 7.4; Bio-Rad) was incubated for 1 h. The secondary antibody (Alexa Fluor 488-conjugated goat anti rabbit IgG, 1:200; Life Technologies, Carlsbad, CA, US) in blocking solution was used to detect the primary antibody. After incubation for 50 min and washing three times with cold 1 × PBS, the cells were mounted with Vectashield mounting medium using 4′,6-diamidino-2-phenylindole (Vector Laboratories, Burlingame, CA, USA), and then visualized using immunofluorescence microscopy (DP72; Olympus, Tokyo, Japan) at 40×.

Rhodamine 123 (Invitrogen, Waltham, MA, USA) was used to measure the membrane polarizations within mitochondria _ENREF_39. Treated cells were stained using 10 mM fluorescent dye for 15 min at 33 °C under 5% CO_2_ for 0 h, 1 h, and 2 h after laser irradiation, and were then washed twice with DMEM without FBS or phenol red (Corning, Tewksbury, MA, US). The relative levels of green fluorescence in each sample were measured with a confocal microscope with emission at 488 nm under equivalent conditions (Leica510 Meta; Carl Zeiss, Oberkochen, Germany). To assess the intensity of fluorescence, the MMP intensity value within the region of interest was measured using ImageJ software (Supplementary Fig. 6) (number of replicates for each experiment was three).

### Statistical analyses

All data were analyzed using GraphPad Prism (GraphPad Software, La Jolla, CA, USA) or SPSS Statistical Software for Windows (IBM, Armonk, NY, USA). The Kolmogorov–Smirnov test was used to determine whether the data were parametric or nonparametric. Significant differences between the control and treatment groups were statistically analyzed using the *t*-test for parametric distributions and the Mann–Whitney U-test for nonparametric distributions, as well as analysis of variance using Tukey’s multiple comparison test and two-way analysis of variance using the Bonferroni post hoc test. A value of *p* < 0.05 was considered statistically significant, and significance is noted as *p < 0.05; **p < 0.01; and ***p < 0.001.

## Supplementary information

Supplementary Figures
