# Supplementary material for: Enhanced mitochondrial membrane potential and ATP synthesis by photobiomodulation increases viability of the auditory cell line after gentamicin-induced intrinsic apoptosis
Source: Sci Rep. 2019 Dec 17;9:19248. doi: 10.1038/s41598-019-55711-9 (PMC6917700; doi:10.1038/s41598-019-55711-9)

**Enhanced mitochondrial membrane potential and ATP synthesis by photobiomodulation increases viability of the auditory cell line after gentamicin-induced intrinsic apoptosis**

**^†^**So-Young Chang^1^, **^†^**Min Young Lee^1, 2^, Phil-Sang Chung^1, 2^, Sehwan Kim^3^, Bernard Choi^4^, Myung-Whan Suh^5^, Chung-Ku Rhee^2^, Jae Yun Jung^1,2rm*^

^1^Beckman Laser Institute Korea, Dankook University, 119, Dandae-ro, Dongnam-gu, Cheonan, Chungnam 31116, Republic of Korea

^2^Department of Otolaryngology-Head and Neck Surgery, College of Medicine, Dankook University, 119, Dandae-ro, Dongnam-gu, Cheonan, Chungnam 31116, Republic of Korea

^3^Department of Biomedical Engineering, College of Medicine, Dankook University, 119, Dandae-ro, Dongnam-gu, Cheonan, Chungnam 31116, Republic of Korea; Vice Director, Beckman Laser Institute Korea, Dankook University, Republic of Korea, Joint appointment in the Beckman Laser Institute at UC, Irvine, CA 92612 USA

^4^Beckman Laser Institute and Medical Clinic, University of California, Irvine, CA 92612 USA

^5^Department of Otorhinolaryngology-Head and Neck Surgery, Seoul National University Hospital, 101 Daehak-ro, Jongno-gu, Seoul 03080, Republic of Korea

**^†^:** These authors contributed equally.

**Corresponding Author**: Jae Yun Jung, MD, PhD., Department of Otolaryngology-Head and Neck Surgery, College of Medicine, Dankook University, 119, Dandae-ro, Cheonan-si, Chungnam, 31116, Republic of Korea, Office: 82-41-550-3973; Fax: 82-41-559-7838; Email: jjkingy2k@gmail.com

**Competing interests:** The authors have no conflicts of interest to declare.

**Supplementary Figure 1. Full-length blots of intrinsic pathway proteins.**

**(A): Cyt c (Cytosol), (B): Cleaved Caspase 9, (C): Cleaved Caspase 3, (D): β-actin**

**
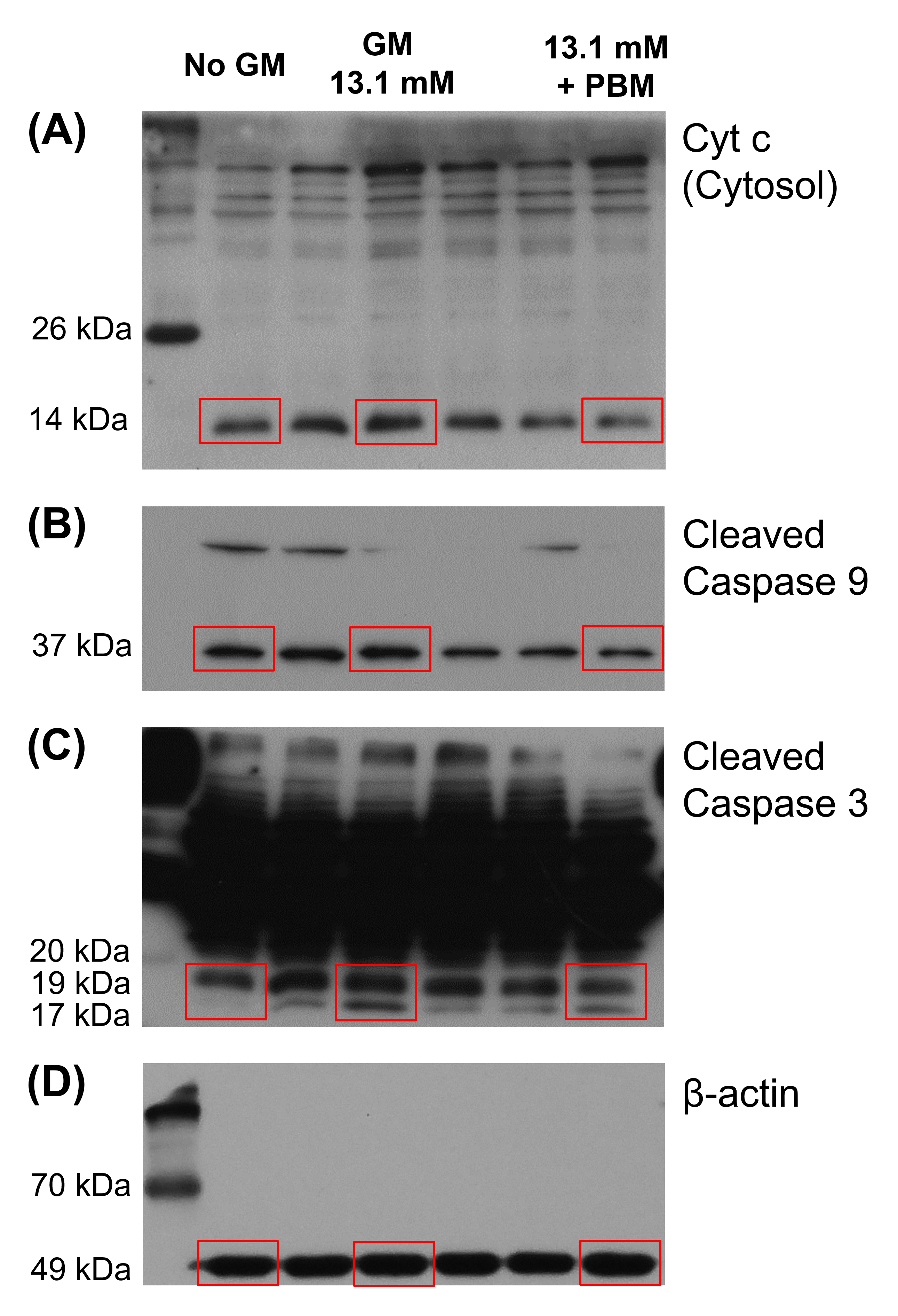
**

**Supplementary** **Figure 2**. **Antimycin A concentration and exposure time-dependent changes in cell viability.**

**
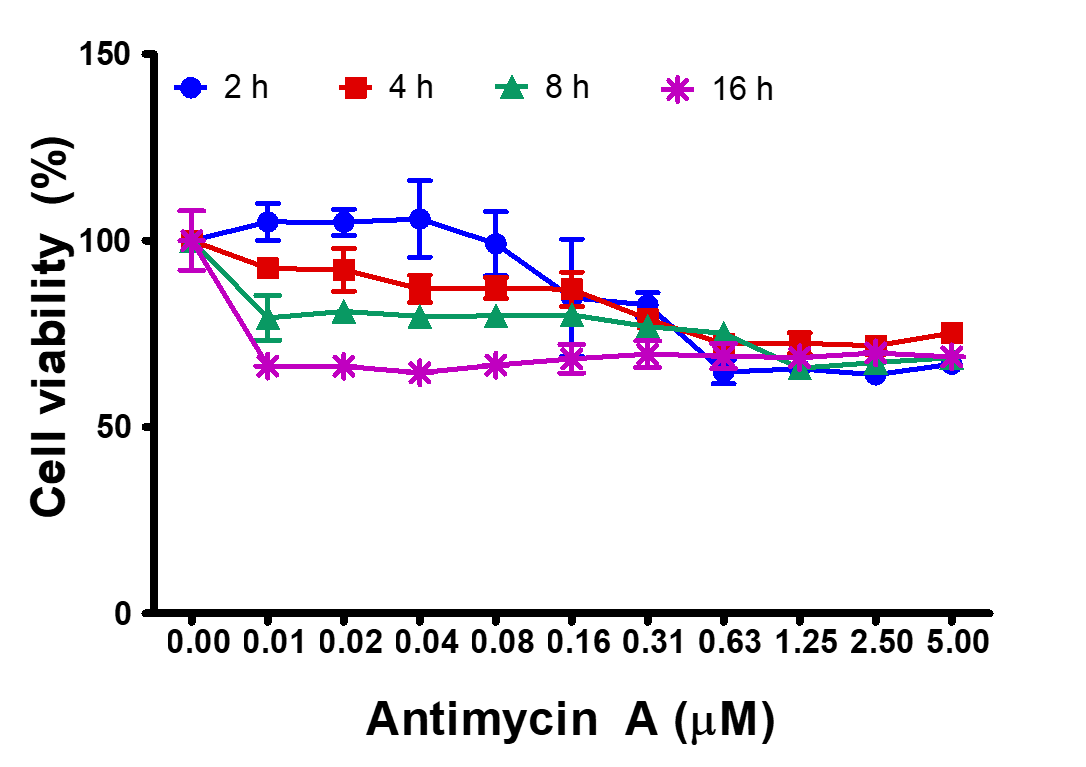
**

**Supplementary Figure 3. Effect of PBM in damaged and non-damaged HEI-OC1 cells. (A): Cell viability, (B): ATP level, (C): MMP intensity. [Two-tailed *t*-test and the Mann–Whitney U-test, p =*: <0.05, **: <0.01, ***: <0.001].**


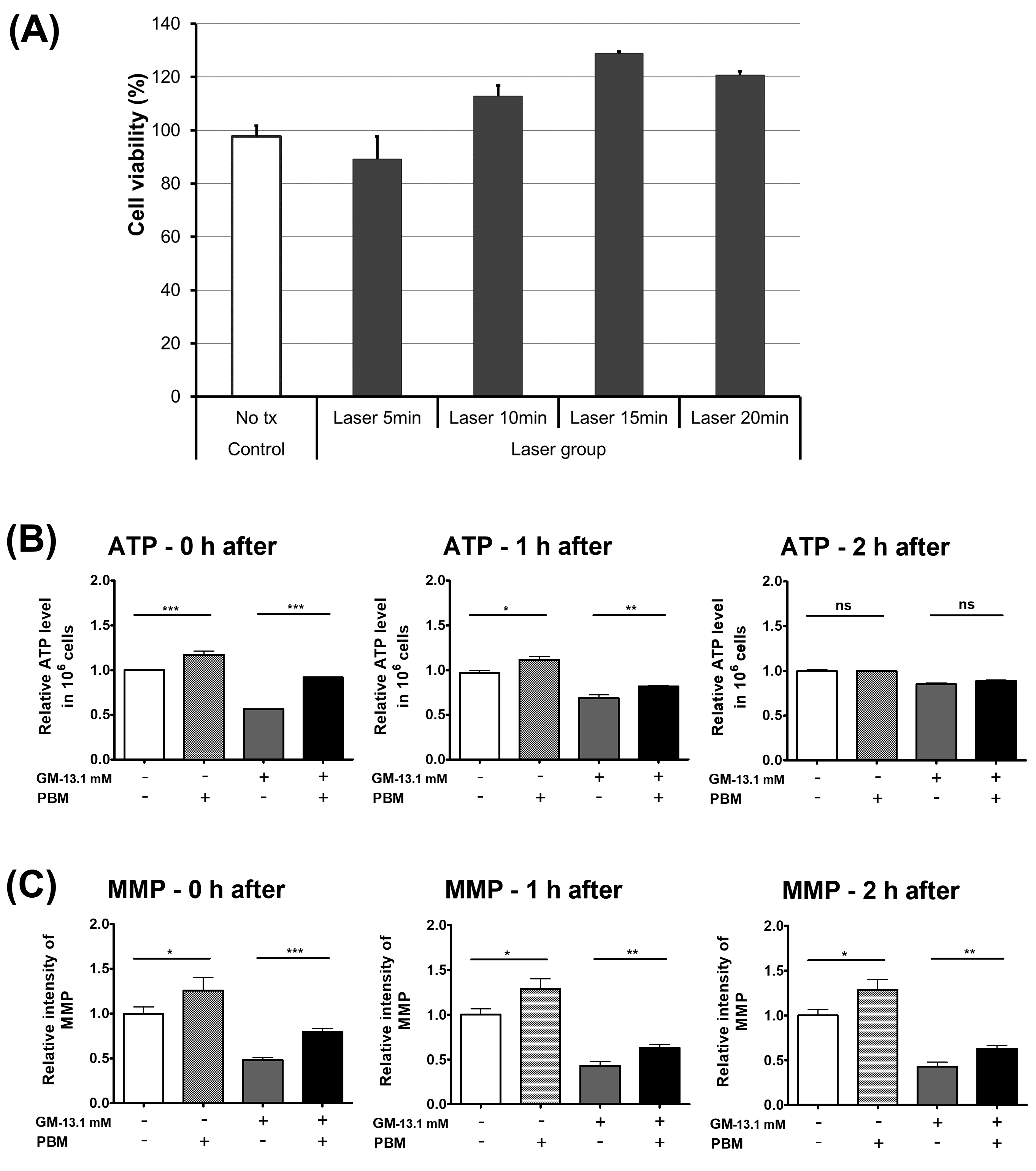


**Supplementary Figure 4. Differences in cell viability after photobiomodulation of varying duration [one-way ANOVA, p = *: <0.05, ***: <0.001 (Tukey’s Multiple Comparison Test)].**


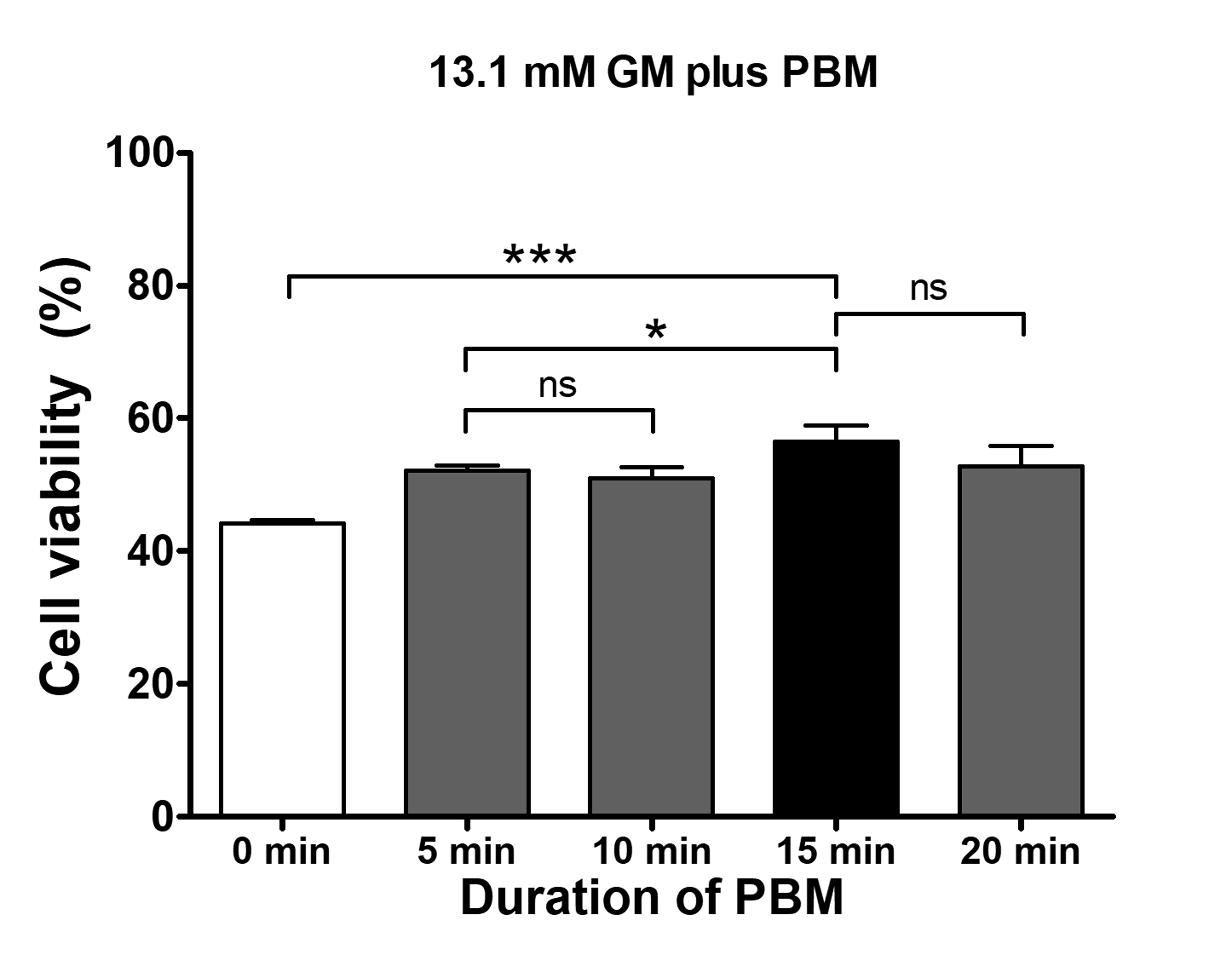


**Supplementary Figure 5. Difference of ATP level between PBM and no PBM in both GM and no GM group, showing significant reduction of ATP level in GM group over time [Mann–Whitney U-test, two-tailed, n = 8, p = 0.003 **: <0.01].**

**
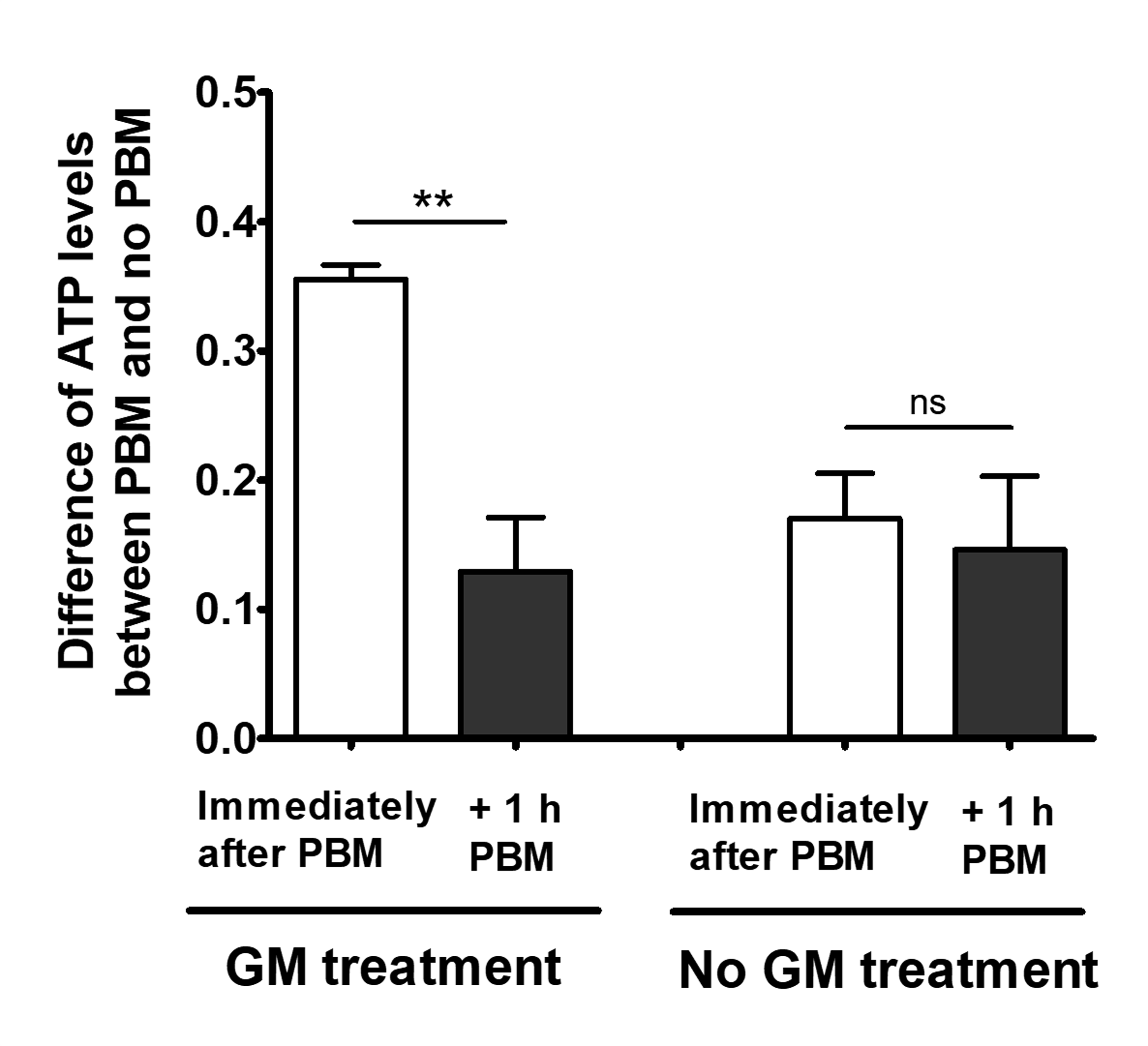
**

**Supplementary Figure 6. Measurement of epifluorescence intensity in HEI-OC1.**


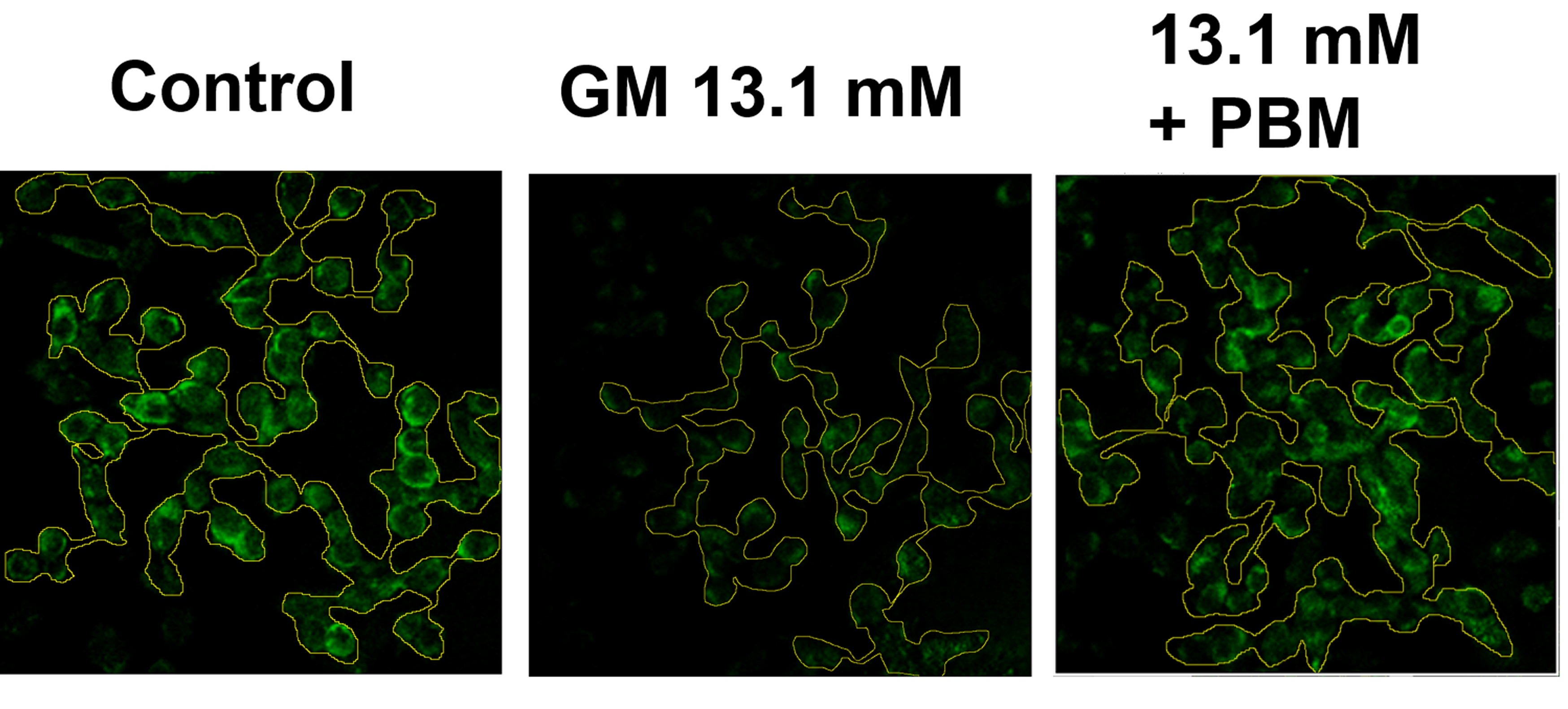

Supplement: Supplementary file 1 — Supplementary Figures [file 41598_2019_55711_MOESM1_ESM.doc]
